# Health-Related Quality of Life (HRQoL) in Idiopathic Inflammatory Myopathy: A Systematic Review

**DOI:** 10.1371/journal.pone.0160753

**Published:** 2016-08-09

**Authors:** Valérie Leclair, Malin Regardt, Sophie Wojcik, Marie Hudson, Canadian Inflammatory Myopathy Study (CIMS)

**Affiliations:** 1 Department of Medicine, Division of Rheumatology, McGill University, Montreal, Canada; 2 Department of Occupational Therapy, Karolinska University Hospital, Stockholm, Sweden; 3 Department of Learning, Division of Informatics and Medical Education, Karolinska Institutet, Stockholm, Sweden; 4 Department of Medicine, Division of Rheumatology, Jewish General Hospital, Montreal, Canada; 5 Lady Davis Institute for Medical Research, Division of Clinical Epidemiology, Jewish General Hospital, Montreal, Canada; JAPAN

## Abstract

Health-related quality of life (HRQoL) is a research priority in chronic diseases. We undertook a systematic review (registration #CRD42015024939) to identify, appraise and synthesize the evidence relating to HRQoL in idiopathic inflammatory myopathies (IIM). A comprehensive search was conducted in August 2015 using CINAHL, EMBase and Pubmed to identify studies reporting original data on HRQoL in IIM using generic HRQoL instruments. Characteristics of samples and results from selected studies were extracted and appraised using a standardized approach. Qualitative synthesis of the results was performed. Ten studies including a total of 654 IIM subjects were included in this systematic review. HRQoL was significantly impaired in all subsets of IIM compared with the general population. Disease activity, disease damage and chronic disease course were associated with poorer HRQoL. Insufficient or conflicting results were found in associations between clinical features, treatment, disease duration and mood or illness perception, and HRQoL in IIM. This study suggests that HRQoL is impaired in IIM. However, due to the paucity and heterogeneity of the evidence to date, robust estimates are lacking and significant knowledge gaps persist. There is a need for studies that systematically investigate the correlates and trajectory of HRQoL in IIM.

## Introduction

Patient-reported outcomes (PROs), including health-related quality of life (HRQoL), are important outcomes in chronic diseases. Idiopathic inflammatory myopathy (IIM) are a group of chronic autoimmune diseases characterized by muscle inflammation and weakness [1]. However, IIM are rare, heterogeneous diseases that lack a standard nomenclature. The widely used Bohan & Peter classification criteria divide adult IIM as follows: dermatomyositis (DM), polymyositis (PM), DM or PM associated with neoplasia and DM or PM associated with connective tissue diseases [2, 3]. Sontheimer subsequently defined two other DM subsets: clinically amyopathic DM (CADM) and hypomyopathic DM [4]. A more recent classification adopted at the European Neuromuscular Centre Workshop (ENMC) in 2003 is also increasingly used and introduced two new entities: necrotizing autoimmune myopathy (NAM) and nonspecific [5]. Inclusion body myositis (IBM), also considered an IIM, has specific clinicopathologic features and separate classification criteria [6].

HRQoL is a complex concept that can be defined as someone’s perception of their physical health, psychological state, level of independence, social relationships and relationship to salient features of their environment [7]. Patients with chronic muscle diseases have compromised HRQoL compared with the general population when assessed with generic and disease-specific instruments [8]. One of the most widely used generic HRQoL instrument, the Medical Outcomes Study 36-items Short Form (SF-36), has shown good construct and content validity in adult DM, PM, and IBM patients [9]. Other generic instruments have also been used, although, to our knowledge, they have not been formally validated for this population [10–12]. There are no disease-specific HRQoL instruments for IIM, but skin-specific tools like the Skindex and the Dermatology Life Quality Index (DLQI) have frequently been used in DM [9]. Studies on HRQoL in IIM to date have generally reported on small, at times selected samples. The objective of this study was to perform a systematic review of the literature in order to generate robust estimates of the magnitude and correlates of HRQoL impairment in IIM patients.

## Materials and Methods

We undertook this systematic review using a pre-defined protocol registered in the PROSPERO database (#CRD42015024939) and followed the preferred reporting items for systematic reviews and meta-analyses (PRISMA) statement [13].

### Eligibility criteria

#### Types of studies

We screened full-length manuscripts and meeting abstracts without language restrictions. Due to the high risk of selection bias in case reports, we selected only published, full-length manuscripts with all types of study design but reporting on at least 20 or more study subjects.

#### Types of participants

All participants were over 18 years of age and had one of the following diagnoses: dermatomyositis (DM), polymyositis (PM), amyopathic dermatomyositis, hypomyopathic dermatomyositis, cancer-associated myositis, connective tissue disease-associated myositis (also known as overlap myopathy (OM)) or inclusion body myositis (IBM). Studies including a mixed population were included provided that data specific to one or more of these diagnoses was presented separately.

#### Types of outcome measures

The main outcome was HRQoL measured using any generic HRQoL instrument. Studies included had to provide HRQoL scores, which in general are means and standard deviations for the whole or subsets of the population. Authors of original articles were contacted to provide missing scores if HRQoL measurement was mentioned in their article, but the data was not shown or incomplete.

### Information sources and search

Pubmed, EMBase and CINAHL were searched from database inception to August 18, 2015. The complete search strategy, which was developed with the assistance of a professional librarian, can be found in S1 Table. Reference lists of eligible articles were screened for other potentially relevant article titles. Conference abstracts were used to track full-length published articles.

### Study selection

Initial screening of titles and abstracts to identify eligible studies was performed by two independent reviewers (VL, SW) in an unblinded manner. We excluded studies on pediatric populations, those reporting on < 20 IIM subjects, not using a generic or IIM-specific HRQoL questionnaire or not reporting separate IIM HRQoL data. All studies selected by either reviewer was included in the next stage of the review. Full-length papers of selected articles were screened by one reviewer (VL), who proceeded to the final selection of eligible studies.

### Data extraction

One reviewer (VL) proceeded to data extraction using a specifically designed extraction form adapted from the Cochrane Consumers and Communication Review Group’s data extraction template. Eight authors of studies included in the review were contacted by email for missing data, consisting mostly of versions of tools used, demographic characteristics of subjects and numerical scores of HRQoL. A second, reminder email was sent after one month if no answer was received to the first request.

### Data items

The following data were extracted from the selected articles: 1) study identification and characteristics (including design, method of recruitment, inclusion / exclusion criteria, sample size, classification criteria used to characterize study subjects, and statistical approach), 2) participant characteristics (including setting, age, sex, ethnicity, IIM subset and severity, disease duration, comorbidities, and treatment), 3) intervention, if applicable, 4) outcomes (including HRQoL instruments used and numerical scores, timing of assessment, and correlates of HRQoL).

### Risk of bias assessment

Seven of the 10 studies included in the review were cross-sectional, non-interventional, and non-randomized studies [11–12, 14–18]. There is no standard risk of bias tool for such studies. They are usually considered case series at high risk of bias [19]. One study pooled the baseline data of 2 randomized controlled trials [20] and as such was also considered a case series. Two studies were cohort studies [10, 21] and risk of bias of these studies was assessed using A Cochrane Risk of Bias Assessment Tool: for Non-Randomized Studies of Interventions (ACROBAT-NRSI) [22].

### Statistical analysis

Data were extracted, summarized in tabular form and synthesized qualitatively. Paucity, heterogeneity and poor quality data precluded pooled, quantitative analysis.

## Results

### Study selection

**Fig 1:**
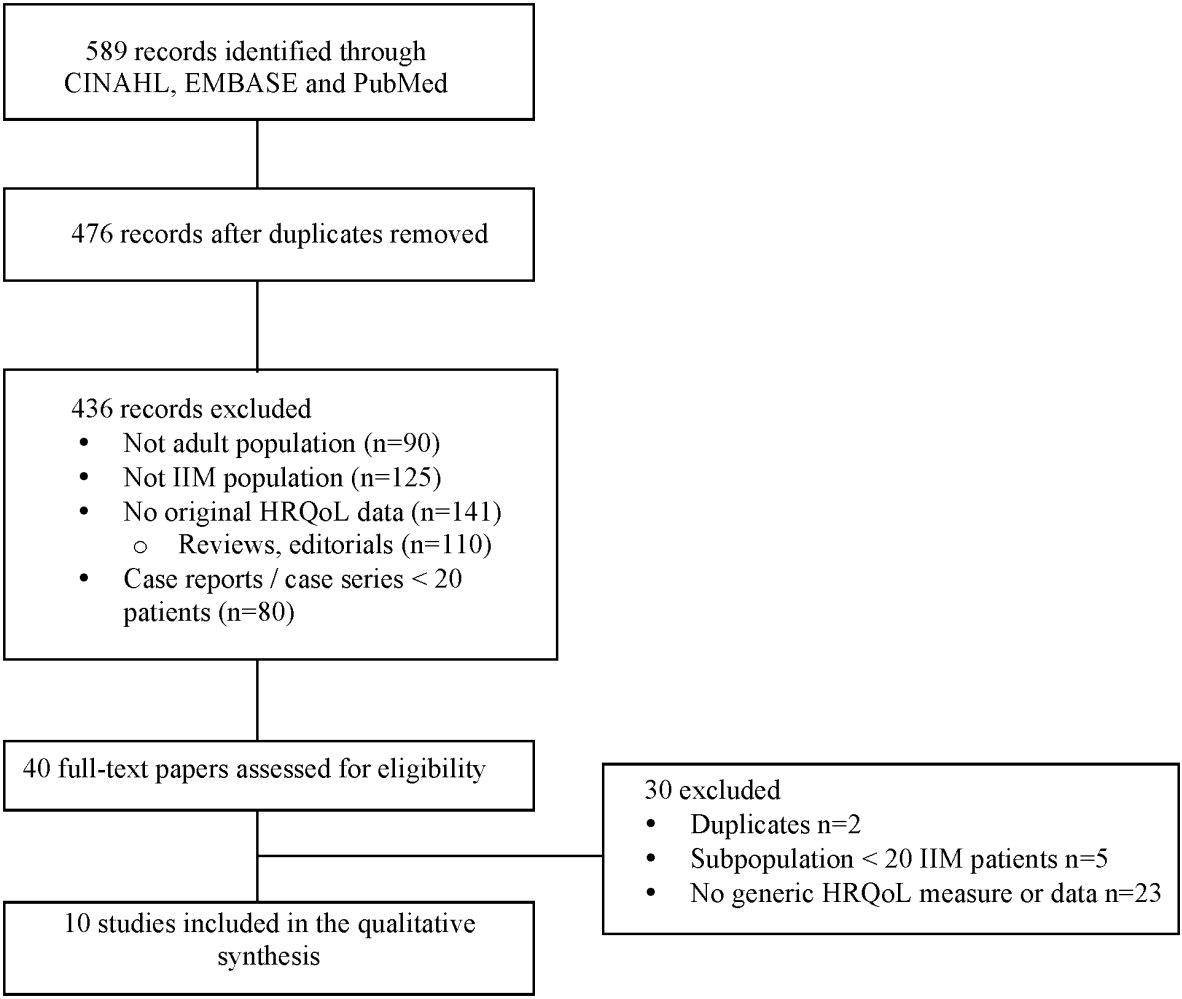
Flow diagram of study selection.

The electronic search identified 589 potentially relevant articles (Fig 1). After removal of duplicates (n = 113) and ineligible papers based on title and abstract review (n = 436), 40 manuscripts were selected for full-text review. Thirty studies were excluded at this stage: 23 because they did not report original data on IIM subjects using a generic HRQoL measure, 5 reported on <20 IIM subjects and 2 reported data that had been previously published in studies already selected for the review [23,24]. No additional studies were identified through reference lists of selected articles or review of conference abstracts. Thus, 10 studies [10–12, 14–18, 20, 21] including 654 IIM subjects (range 31–113) in whom HRQoL was measured using a generic questionnaire were included in this review. Two authors generously provided missing information including their complete SF-36 results [16, 21].

### Study characteristics

The majority of the studies included were cross-sectional (n = 7) (Table 1). Two studies were cohort studies. One study pooled the baseline results of 2 randomized controlled trials and was included instead of the original papers because those showed either negative results [24] or did not report their SF-36 results [23]. All the studies were from Europe or the US and five used either the Bohan & Peter [12, 16, 17, 19] or the ENMC classification criteria [21] for subject selection. The others did not specify this information. There was considerable heterogeneity in the IIM subsets included in each study, although the majority included DM and PM. The most commonly used HRQoL questionnaire was the SF-36 (n = 7). Four other tools were used: the Sickness Impact Profile (SIP), the Nottingham Health Profile (NHP), the Individualised Neuromuscular Quality of Life Questionnaire (INQoL) and the WHO Quality of Life Measure (WHOQOL-BREF). A short description of each tool is provided in S2 Table.

**Table 1:** Description of the studies included in this review.

Study	Design	Country	QoL Measure	N*/ total	Subset	Age, years†	Female (n)	Disease duration, years†	Follow-up, years†
Armadans [12]	Cross-sectional	Spain	WHOQOL-BREF	62/62	DM, PM	50,7±16,2	52	5,4±4,6	
Bronner [10]	Cohort	Nether-lands	SIP‡	110/165	DM, PM, OM	45±17	120	Ø	6±4,4
Chung [11]	Cross-sectional	UK	NHP	113/113	DM, PM	DM: 50 (25–75) PM: 54 (22–76)	113	DM: 7 (1–26) PM: 7 (1–27)	
Goreshi [14]	Cross-sectional	US	SF-36	52/110	DM	Ø	74	Ø	
Ponyi [15]	Cross-sectional	Hungary	SF-36	87/90	DM, PM, OM	50±10,9	67	Ø	Median 9 (3–23)
Regardt [16]	Cross-sectional	Sweden	SF-36	31/31	DM, PM	56,1±10,6	18	6,8±5,5	
Rose [17]	Cross-sectional	US	SF-36, INQOL	43/302	DM, PM, IBM	Ø	19	Ø	
Sadjadi [20]	RCT	UK	SF-36	60/60	IBM	64,5±8,5	22	4,4±3	
Sultan [18]	Cross-sectional	UK	SF-36	34/46	DM, PM, CDM^§^, OM	52	26	Ø	
van de Vlekkert [21]	Cohort	Nether-lands	SF-36	62/62	DM, OM, NSM, NAM	49±14	39	Median 4 months	3±1,5

WHOQOL-BREF World Health Organization Quality of Life—BREF, SIP Sickness Impact Profile, NHP Nottingham Health Profile, SF-36 Medical Outcomes Study 36-items Short Form, INQOL Individualised Neuromuscular Quality of Life Questionnaire, PM polymyositis, DM dermatomyositis, IBM inclusion body myositis, OM overlap myositis, CDM childhood onset dermatomyositis, NSM non-specific myositis, NAM necrotizing autoimmune myositis, RCT randomized controlled trial.

* number of IIM patients with HRQoL scores.

^†^Values are the mean ± SD or (range), at baseline.

^‡^Only 3 subscales used (body care and movement, walking and mobility).

^Ø^ not reported.

^§^ 1 patient out of the 46 patient had a diagnosis of CDM.

### HRQoL in IIM compared to healthy controls or other chronic diseases

HRQoL scores of IIM subjects were compared with those of the general population in 6 studies [11, 14–16, 18, 22] (Table 2, S3 Table). There was consistent impairment, regardless of the questionnaire used, with a majority of the studies reporting significantly lower scores in all HRQoL domains [11, 15, 16, 18]. Three studies compared IIM with other chronic diseases (Tables 3–5). Chung et al. [11] found PM and DM subjects to have poorer scores in energy and social isolation domains than subjects with other rheumatologic conditions. However, physical disability was similar to rheumatoid arthritis and pain in IIM was less than in rheumatoid arthritis and osteoarthritis. Goreshi et al. [14] compared the SF-36 scores of DM subjects with that of subjects with systemic lupus erythematosus, congestive heart failure, cutaneous lupus erythematosus, depression, recent myocardial infarction, hypertension and diabetes. They reported more impaired HRQoL in IIM in the physical and mental health domains compared with several of those chronic conditions. Rose et al. [17] compared DM, PM and IBM subjects with other chronic neuromuscular diseases using the INQoL and reported comparable reduced scores for all those muscle disorders.

**Table 2:** Summary of HRQoL results using the SF-36.

			Domains (means)								
Studies	Subsets	n	VT	PF	BP	ER	SF	GH	PR	MH	Scoring‡
Sadjadi [20]	IBM	60	47	24	69	76	66	58	39	78	Additive
Goreshi [14]	DM	52	44*	40*	50†	43*	43*	42*	41*	45*	Norm
	**GP**		**52**	**51**	**52**	**49**	**50**	**51**	**51**	**50**	Norm
Ponyi [15]	DM	21	42*	48*	54*	70*	48*	51*	55*	51*	Additive
	PM	52	52*	50*	58*	68*	51*	60*	55*	60*	Additive
	OM	14	42*	38*	66*	68*	41*	42*	41*	48*	Additive
	**GP**		**70**	**91**	**78**	**78**	**80**	**64**	**79**	**71**	Additive
Regardt [16]	DM	11	46*	56*	55*	73*	74*	50*	30*	76*	Additive
	PM	20	41*	45*	59*	63*	59*	43*	26*	69*	Additive
	**GP**		**68**	**82**	**70**	**83**	**88**	**71**	**76**	**80**	Additive
Sultan [18]	DM/PM	34	35*	42*	55*	57*	50*	39*	42*	57*	Additive
	**GP**		**75**	**92**	**90**	**85**	**63**	**85**	**90**	**78**	Additive
van de Vlekkert [21]	DM	23	34	24	37	94	64	31	14	72	Additive
	OM	12	25	33	30	75	61	35	13	60	Additive
	NAM	4	14	13	64	83	50	34	0	41	Additive
	NSM	22	37	37	52	95	77	39	18	65	Additive
	**GP**		**70**	**90**	**80**	**80**	**78**	**70**	**80**	**80**	Additive

PM polymyositis, DM dermatomyositis, IBM inclusion body myositis, GP general population, PF physical functioning, PR physical role, BP bodily pain, GH general health, VT vitality, SF social functioning, ER emotional role, MH mental health.

^‡^For further details on the type of scoring, refer to S2 Table.

* Significant difference from general population (p<0.05) indicated when provided by the authors.

^†^110 patients with BP scores.

**Table 3:** Comparison of HRQoL between IIM and other rheumatologic diseases using the NHP.

			Domains					
Study	Condition	n	Energy	Physical	Pain	Emotion	Social	Sleep
Chung [11]	DM/PM	113	74	42	30	25	25	28
	OA	96	43*	27*	41*	14*	11*	32
	OP	45	32*	22*	33	13*	11*	31
	RA	142	50*	40	49*	28	16*	32

PM polymyositis, DM dermatomyositis, OA osteoarthritis, OP osteoporosis, RA rheumatoid arthritis.

* p<0,05, indicated when provided by the authors.

**Table 4:** Comparison of HRQoL between IIM and other chronic conditions using the SF-36.

			Domains							
Study	Condition	n	VT	PF	BP	ER	SF	GH	PR	MH
Goreshi [14]	DM	52†	44	40	50	43	43	42	41	45
	SLE	65	40	40	43*	40*	38*	36*	39	41
	CHF	216	44	35*	47	44	45	39	38	50*
	CLE	112‡	49*	50*	54	48*	47*	46*	49*	48
	Depression	502	42	45*	45*	36*	39*	42	41	34*
	Recent MI	107	50*	44	51	47	51*	45	43	50*
	HTN	2089	51*	46*	51	48*	51*	47*	46*	52*
	Diabete	541	49*	44	49	48*	49*	43	44	51*

PM polymyositis, DM dermatomyositis, PF physical functioning, PR physical role, BP bodily pain, GH general health, VT vitality, SF social functioning, ER emotional role, MH mental health, SLE systemic lupus erythematosus, CHF chronic heart failure, CLE cutaneous lupus erythematosus, MI myocardial infarction, HTN hypertension.

* p<0,05, indicated when provided by the authors.

^†^49 patients with ER scores.

^‡^110 patients with BP scores.

**Table 5:** Comparison of HRQoL between IIM and other neuromuscular diseases using the INQOL.

			Domains								
Study	Condition	n	Fatigue	Weak-ness	Pain	Emotion	Social	Indep-endence	Activity	Body image	Locking
Rose [17]	PM/DM	19	58	48	70	50	30	40	42	43	43
	IBM	24	55	64	46	41	33	55	58	56	31
	LGMD	91	47	58	45	46	33	62	63	58	45
	FSHD	49	52	60	40	42	29	42	56	59	39
	MD	79	53	55	41	42	26	42	46	50	50
	Misc.	40	53	63	35	44	30	44	47	55	44

PM polymyositis, DM dermatomyositis, IBM inclusion body myositis, LGMD limb girdle muscular dystrophy, FSHD facioscapulohumeral muscular dystrophy, MD myotonic dystrophy, Misc. miscellaneous.

### Impact of disease subsets

Seven of the 10 studies reported HRQoL in different subsets of IIM [10–12, 14–16, 21] (Table 2, S3 Table). The largest of these by Chung et al. [11] reported worse scores in the physical domain of PM subjects compared to DM subjects (47.1 vs 36.5; p = 0,03). However, 4 studies did not find a significant difference between IIM subsets [12, 15–16, 21]. In 2 of those studies [15, 21], OM subjects had poorer HRQoL scores in certain domains, but those results were sparse and inconsistent, precluding further comparison. Bronner et al. [10] compared the SIP results of PM, DM, unspecified myositis and possible myositis. They, however, report only the number of subjects scoring higher then 1,6% in the physical domain of the tool, number representing the mean physical SIP score of the Dutch general adult population. Considering the data reported, their “possible myositis” population had more abnormal physical SIP results than their other subsets. Goreshi et al. [14] compared HRQoL in classic dermatomyositis (CDM) and amyopathic or hypomyopathic dermatomyositis (AHDM) with, again, no significant differences reported between the subsets. However, CDM and AHDM were not clearly defined and the data on subsets were incomplete and only available for 1 center (42 out of 110 subjects).

### Impact of demographic and disease-related characteristics

The patient-related and disease-related characteristics analyzed as possible correlates of HRQoL in IIM are presented in S4 Table. The key findings are as follows. The effect of age on HRQoL in IIM was explored in 2 studies [12, 15] with inconsistent results. The only reported data in both papers were related to its impact on mental health, with Armadans et al. [12] reporting that older subjects had more impairment in this domains while Ponyi et al. [15] found the opposite. Armadans et al. [12], using univariate analysis, showed that subjects with dysphagia had worse physical health (3.05 vs 3.27; p<0.05) and environment (3.45 vs 3.73; p<0.05) domain scores than those without. Ponyi et al. [15], using multiple linear regression models, found that arthralgias and glucocorticoid-related complications correlated with lower SF-36 scores in several SF-36 domains. Of note, Goreshi et al. [14] showed that pruritus, a symptom often overlooked in DM, was associated with poorer HRQoL using skin-specific questionnaires.

Finally, only 3 studies [10, 12, 15] explored associations between myositis-associated or myositis-specific antibodies and HRQoL on a small sample of patients and were thus underpowered. Ponyi et al. [15] found that anti-Jo1 positivity was associated to lower scores in the role emotional domain (r = -18.8, p = 0.032) using the results of 40 subjects with available anti-Jo1 level at diagnosis. Bronner et al. [10] looking at subjects with positive myositis-specific antibodies (anti-Jo1 n = 14, anti-synthetase n = 6, anti-Mi2 = 20, anti-SRP n = 3), reported poorer physical scores in patients with anti-synthetase antibodies when compared with patients without myositis-specific antibodies (n = 56). Armadans et al. [12] found no significant difference between patients with myositis-specific antibodies (anti-Jo1 n = 17, anti-PL12 n = 3, anti-PL7 n = 2) or without in their sample.

### Impact of disease activity and damage

Eight studies reported results on the influence of disease activity or damage on HRQoL in IIM (S4 Table) [10, 12, 14–16, 18, 20, 21]. Not surprisingly, there was evidence suggesting that disease activity, disease damage and functional impairment in IIM correlated with poorer HRQoL scores mostly in the physical and environmental domains, and less consistently, in the emotional domains of the different tools. However there was considerable heterogeneity in measures of disease activity and damage, HRQoL instruments used and statistical approaches. In addition, several studies provided incomplete data or intentionally looked only at the physical component of their HRQoL tool. Of note, the studies reporting no association between disease activity and damage were in general largely underpowered.

### Impact of treatment

Only two studies [12, 15] reported their results on the influence of treatment on HRQoL. Ponyi et al. [15] reported a positive correlation between initial treatment and mental health. However, no further information was provided on the type of treatment received. Armadans et al. [12] found no association between the number of current immunosuppressive drugs (including corticosteroids and IVIg), and HRQoL. However, the sample was small and heterogeneous, treatment was not standardized and immunosuppressive therapies were pooled together precluding definitive conclusions.

### Impact of disease duration and clinical course

Three studies [12, 20, 21] explored the impact of disease duration on HRQoL and two [12, 20] found no association. However, these used data from cross-sectional assessments, included only a small number of patients (n = 60, n = 62) with a relatively short mean disease duration (4.35 and 5.35 years). The third by Van de Vlekkert et al. [21] found that QoL improved in the first 18 months from diagnosis and then plateaued. Clinical course was examined in 3 studies [15, 18, 21]. Sultan et al. [18] found chronic-progressive disease to be associated with significantly worse bodily pain scores on the SF-36 than relapsing-remitting disease. Van de Vlekkert et al. [21], with a mean follow-up duration of 3 years (sd 1,5), showed reduced SF-36 physical, vitality and general health domain scores in subjects with ≥2 relapses compared to those with a monophasic course. The largest study (n = 87) by Ponyi et al. [15] found no influence of disease course on HRQoL in DM, PM and OM patients with a median follow-up duration of 8.9 years (range 3.0–22.8).

### Impact of mood and illness perception

The impact of depression on HRQoL was assessed by Sadjadi et al. [20] using the Beck Depression Inventory. According to the authors, the significant association between disease activity and SF-36 domains they found was significantly reduced when depression was introduced as a mediating variable. Unfortunately, the data were no longer available to ascertain the magnitude of this association.

### Risk of bias

Seven of the 10 studies included in this review were cross-sectional case series and as such are considered at high risk of bias [13]. The data on HRQoL in the study by Sadjadi et al. [20], collected before randomisation, were also considered cross-sectional and non-controlled. Hence, it too was at high risk of bias. The quality of the two cohort studies [10, 21] was assessed with the Cochrane Collaboration’s tool for assessing risk of bias in non-randomised studies. Using this tool, both were considered at high risk of bias mostly because of selection and reporting bias as well as loss to follow-up [10]. Thus, to date, most studies of HRQoL in IIM are at high risk of biases, including selection, non-response, volunteer, survivorship and reporting bias.

## Discussion

In this systematic review of 10 studies including 654 subjects with IIM, HRQoL was impaired compared to the general population and subjects with other chronic rheumatic and non-rheumatic diseases. This finding was fairly consistent, regardless of the IIM subsets or the HRQoL questionnaire used. This is keeping with the current knowledge on HRQoL in chronic muscle disease [8]. However, there was a paucity of data and the studies included in the review had important limitations including, among others, small, single-center, heterogeneous samples, inconsistent definitions of disease and clinical variables, variable HRQoL measures, incomplete data reporting, statistical approaches that did not control for possible confounding and high risk of bias. This precluded pooling of results to generate robust quantitative estimates of HRQoL in IIM.

Although impaired HRQoL is to be expected in IIM, IIM have particular features that could impact HRQoL differently compared to other chronic diseases and thereby affect the magnitude of impairment. Indeed, IIM can have unpredictable clinical course and significant extra-muscular manifestations. For example, interstitial lung disease, increasingly recognized as an important feature of IIM and associated with higher morbidity and mortality [25], was generally not considered in the studies included in this review. Similarly, dysphagia was considered in only one study [12]. In oculopharyngeal muscular dystrophy, severity of dysphagia has been found to be an independent predictor of the mental component of the SF-36 [26]. Finally, many available treatments options for IIM have important toxicities. Unfortunately, the impact of treatment on HRQoL remains under-studied in IIM.

Disease duration was considered a potential correlate of HRQoL in IIM in only 3 studies. Longer duration has been found to correlate with poorer HRQoL in other chronic diseases [8]. Another important aspect to consider is disease course, especially with some of the studies reviewed here showing worse HRQoL with relapsing-remitting or chronic courses. It is however difficult to determine the exact effect of disease course on HRQoL based on cross-sectional studies, but we can hypothesize that it follows different trajectories depending on disease evolution. In early monophasic disease HRQoL is probably worse at diagnosis with improvement on treatment, in opposition to HRQoL gradually worsening in a chronic progressive course. Large studies of incident subjects with adequate representation of each subset and long follow-up will be required to fill this important knowledge gaps.

We would also like to point the lack of data concerning the impact of mood and disease perception on HRQoL. Some evidence reported here suggests impairment of HRQoL in psychological/emotional domains of the different tools in all subsets of IIM and a possible mediating role of anxiety and depression on HRQoL. This important aspect should also be further investigated as it has the potential to modulate HRQoL regardless of the chronic physical or functional impairments of a particular IIM subject.

Comparing heterogeneous populations classified according to different criteria with a lack of reported diagnostic methods was challenging. Only one study found a difference between HRQoL in DM and PM patients. This may be the result of selection bias with studies including exclusively outpatient populations, possibly less severe cases, and often excluding certain subsets. Indeed, IBM, OM and cancer-associated myositis were only a small fraction of the patients included in the studies. The Bohan and Peter criteria were developed before IBM was described, which might lead to misclassification of IBM as PM patients [27]. The lack of difference between subsets could also be explained by survivorship bias, with over-representation of milder cases with longer disease duration or follow-up time, homogenizing the results obtained. In a recent Chinese study, the first peak of mortality in their IIM cohort was at 3 years [28]. In our review, the studies reporting on different IIM subsets and disease duration had a mean disease duration of > 5 years. Yet again, this shows that cross-sectional studies on IIM subjects with established disease are not suited to answer the question of HRQoL in early disease and its trajectory over time.

Finally, while it is clearly beyond the scope of this paper to compare the strengths and limitations and the performance characteristics of the various HRQoL tools available, our study clearly indicates that additional standardized research will be critical to generate robust estimates of HRQoL in IIM. Thus, for the time being, we would align ourselves with IMACS which suggests using the original SF-36 in adult myositis clinical trials and studies (http://www.niehs.nih.gov/research/resources/imacs/patientoutcome/index.cfm). In the future, the development and validation of myositis-specific HRQoL tool(s) would be needed to gain a higher level of granularity in the understanding of the impact of specific disease features and subsets of IIM on HRQoL.

### Limitations

This study is not without some limitations. First, in order to facilitate comparisons in general HRQoL, we excluded studies using symptom-specific HRQoL tools. Thus, some aspects of HRQoL in IIM could have been under-represented in this review (ex: skin manifestation). Second, we also excluded studies reporting on < 20 subjects. This represented approximately 118 IIM subjects. However, small case series are at high risk of bias, in particular selection bias, thereby compromising the generalizability of their findings. Third, the significant heterogeneity in the available data precluded pooled, quantitative analysis

## Conclusions

In this systematic review of the literature, we found some evidence that HRQoL is impaired in IIM. However, due to the paucity and heterogeneity of the evidence to date, robust estimates are lacking and significant knowledge gaps persist. There is a need for studies that systematically investigate the trajectory and factors associated with HRQoL in IIM. A better understanding of the magnitude and correlates of impaired HRQoL in IIM is a sine qua non to the development of interventions to improve HRQoL in IIM.

## Supporting Information

S1 Checklist PRISMA checklist. (DOC)

S1 Table Search strategies. (PDF)

S2 Table Description of the generic HRQoL tools used in selected studies. (DOCX)

S3 Table Summary of HRQoL results of the studies included in the review. (DOCX)

S4 Table Impact of clinical characteristics on HRQoL. (DOCX)
