# Supplementary material for: Health-Related Quality of Life (HRQoL) in Idiopathic Inflammatory Myopathy: A Systematic Review
Source: PLoS One. 2016 Aug 9;11(8):e0160753. doi: 10.1371/journal.pone.0160753 (PMC4978480; doi:10.1371/journal.pone.0160753)
Supplement: S1 Table — (PDF) [file pone.0160753.s002.pdf]

S1 Table - Search strategies

Database	Search strategies
**PubMed**	((((("Myositis"[Mesh:noexp]) OR "Dermatomyositis"[Mesh]) OR "Myositis, Inclusion Body"[Mesh]) OR "Polymyositis"[Mesh] OR "inflammatory myopathy" OR "inflammatory muscle disease" OR "idiopathic inflammatory myopathy" OR "myositis" OR "polymyositis" OR "dermatomyositis" OR "inclusion body myositis")) AND ((("Quality of Life"[Mesh]) OR "Sickness Impact Profile"[Mesh] OR "Medical Outcomes Study Short Form 36" OR "SF-36" OR "WHOQOL-BREF" OR "Medical Outcomes study short form 12" OR "SF-12" OR "Nottingham Health Profile" OR "Sickness impact profile" OR "Medical Outcomes study short form 6D" OR "SF-6D" OR "Health Utilities Index Mark 3" OR "HUI3" OR "Quality of Well-being" OR "QWB" OR "Assessment of quality of life" OR "well-being"[OT] OR "quality of life"[OT]))
**EMBASE**(OvidSP)	1. myositis/ or antisynthetase syndrome/ or dermatomyositis/ or inclusion body myositis/ or polymyositis/<br>2. (inflammatory myopathy or inflammatory muscle disease or idiopathic inflammatory myopathy or polymyositis or dermatomyositis or myositis or inclusion body myositis or antisynthetase syndrome or anti-synthetase syndrome).mp. [mp=title, abstract, heading word, drug trade name, original title, device manufacturer, drug manufacturer, device trade name, keyword]<br>3. 1 or 2<br>4. "quality of life"/ or "quality of life index"/ or short form 36/<br>5. short form 36/ or short form 12/ or short form 20/ or short form 8/<br>6. sickness impact profile/<br>7. WHOQOL-BREF.mp.<br>8. nottingham health profile/<br>9. Quality of Well-Being scale.mp.<br>10. Assessment of Quality of Life.mp.<br>11. (SF-36 or SF-12 or SF-6D).mp. [mp=title, abstract, heading word, drug trade name, original title, device manufacturer, drug manufacturer, device trade name, keyword]<br>12. Sickness impact profile.mp.<br>13. quality of life.mp.<br>14. nottingham health profile.mp.<br>15. 4 or 5 or 6 or 7 or 8 or 9 or 10 or 11 or 12 or 13 or 14<br>16. Health Utilities Index.mp.<br>17. 15 or 16<br>18. 3 and 17
**CINAHL (EBSCOHost)**	S8 S3 AND S7<br>S7 S4 OR S5 OR S6<br>S6 SF-36 OR SF-12 OR SF-6D OR SF-6 OR Nottingham Health Profile OR Sickness impact profile OR Quality of Well-being OR Health Utilities Index OR Assessment of quality of life OR quality of life<br>S5 (MH "Short Form-36 Health Survey (SF-36)") OR (MH "Sickness

	Impact Profile")<br>S4 (MH "Quality of Life") OR (MH "Comfort") OR (MH "Occupation (Human)") OR (MH "Quality of Working Life")<br>S3 S1 OR S2<br>S2 Myositis OR Antisynthetase Syndrome OR Dermatomyositis OR "inclusion body myositis" OR Polymyositis<br>S1 (MH "Myositis") OR (MH "Antisynthetase Syndrome") OR (MH "Dermatomyositis") OR (MH "Myositis, Inclusion Body") OR (MH "Polymyositis")
