# Supplementary material for: Health-Related Quality of Life (HRQoL) in Idiopathic Inflammatory Myopathy: A Systematic Review
Source: PLoS One. 2016 Aug 9;11(8):e0160753. doi: 10.1371/journal.pone.0160753 (PMC4978480; doi:10.1371/journal.pone.0160753)
Supplement: S2 Table — (DOCX) [file pone.0160753.s003.docx]

**S2 Table - Description of the generic HRQoL tools used in selected studies**

| **Tool** | **Domains** | **Scoring** |
| --- | --- | --- |
| **Medical Outcomes Study 36-items Short Form (SF-36)** | **8 domains**: physical functioning, role-physical, bodily pain, social functioning, general mental health, role-emotional, vitality, general health perception | Profile of 8 section scores + 2 summary scores (physical component (PCS) and mental component (MCS) summary scores). 2 scoring approaches :   - additive (0 to 100 score) for each scale (MCID 5-10) - norm-based approach with adjustment of raw scores to have a mean of 50 and a sd of 10 (MCID 2-5) |
| **WHOQOL-BREF** | **6 domains :** overall QoL, overall general health, physical health, psychological health, social relations, environment. | Individual scores using five-point Likert-type scales for each domains, with higher scores indicating a poorer QoL.  MCID not established. |
| **Sickness Impact Profile (SIP)** | **3 dimensions** (physical, psychosocial and independent categories) divided in **12 categories** (ambulation, mobility, body care and movement, communication, alertness behavior, emotional behavior, social interaction, sleep and rest, eating, work, home management, recreation and pastimes. | Each category may be scored separately, or 2 dimension scores may be formed:   - physical score (ambulation, mobility, and body care and movement) - psychosocial score (social interaction, alertness, emotional behavior, and communication)   MCID not established. |
| **Nottingham Health Profile (NHP)** | **Part I**: 6 sections including physical abilities, pain, sleep, social isolation, emotional reactions and energy level  **Part II**: seven items covering the effect of health problems on occupation, jobs around the house, personal relationships, social life, sex life, hobbies, and holidays. | Yes or no response to each item with a weighted score yielding a result between 0 (no problem) and 100 (all items checked) for each scale.  MCID not established. |
| **Individualised Neuromuscular Quality of Life Questionnaire (INQoL)** | **10 domains**:   - 4 symptoms: weakness, fatigue, pain, locking - 5 life domains: activities, independence, social, emotional and body image. | Each domain reported separately in percentage (%) with a higher (%) indicating poorer QoL. The 5 life domains can be combine to calculate an overall INQoL score.  MCID not established. |

Legend: sd standard deviation, QoL quality of life

MCID: minimum clinically important difference
