# Supplementary material for: Health-Related Quality of Life (HRQoL) in Idiopathic Inflammatory Myopathy: A Systematic Review
Source: PLoS One. 2016 Aug 9;11(8):e0160753. doi: 10.1371/journal.pone.0160753 (PMC4978480; doi:10.1371/journal.pone.0160753)
Supplement: S3 Table — (DOCX) [file pone.0160753.s004.docx]

**S3 Table - Summary of HRQoL results of the studies included in the review**

| **Study** | **Subsets** | **n** | **Domains (mean)** | | | | | | | |  |
| --- | --- | --- | --- | --- | --- | --- | --- | --- | --- | --- | --- |
| **SIP** |  |  | **Physical >1.6%**  **n (%)** |  |  |  |  |  |  |  |  |
|  |  |  |  |  |  |  |  |  |  |  |  |
| Bronner [10] | PM | 6 | 5 (83) |  |  |  |  |  |  |  |  |
|  | DM | 41 | 33 (80) |  |  |  |  |  |  |  |  |
|  | Unspecified. | 40 | 33 (83) |  |  |  |  |  |  |  |  |
|  | Possible‡ | 23 | 22 (96) |  |  |  |  |  |  |  |  |
| **WHOQOL**  **BREF£** |  |  | **WHO-5 scale** | **Physical health** | **Environ-ment** | **Psychol-ogical** | **Social relations** |  |  |  |  |
| Armadans [12] | DM | 47 | 3.27 | 3.22 | 3.6 | 3.36 | 3.74 |  |  |  |  |
|  | PM | 15 | 3.05 | 2.96 | 3.51 | 3.24 | 3.8 |  |  |  |  |
| **NHP** |  |  | **Energy** | **Physical** | **Pain** | **Emotion** | **Social** | **Sleep** |  |  |  |
| Chung [11] | DM | 57 | 74 | 37§ | 30 | 28 | 26 | 26 |  |  |  |
|  | PM | 56 | 74 | 47§ | 31 | 22 | 25 | 30 |  |  |  |
|  | GP |  | 14 | 7 | 6 | 10 | 7 | 16 |  |  |  |
| **SF-36** |  |  | **VT** | **PF** | **BP** | **ER** | **SF** | **GH** | **PR** | **MH** | **Scoring** |
| Sadjadi [20] | IBM | 60 | 47 | 24 | 69 | 76 | 66 | 58 | 39 | 78 | Additive |
| Goreshi [14] | DM | 52 | 44* | 40* | 50† | 43* | 43* | 42* | 41* | 45* | Norm |
|  | **GP** |  | **52** | **51** | **52** | **49** | **50** | **51** | **51** | **50** |  |
| Ponyi [15] | DM | 21 | 42* | 48* | 54* | 70* | 48* | 51* | 55* | 51* |  |
|  | PM | 52 | 52* | 50* | 58* | 68* | 51* | 60* | 55* | 60* |  |
|  | OM | 14 | 42* | 38* | 66* | 68* | 41* | 42* | 41* | 48* |  |
|  | **GP** |  | **70** | **91** | **78** | **78** | **80** | **64** | **79** | **71** | Additive |
| Regardt [16] | DM | 11 | 46* | 56* | 55* | 73* | 74* | 50* | 30* | 76* |  |
|  | PM | 20 | 41* | 45* | 59* | 63* | 59* | 43* | 26* | 69* |  |
|  | **GP** |  | **68** | **82** | **70** | **83** | **88** | **71** | **76** | **80** | Additive |
| Sultan [18] | DM/PM | 34 | 35* | 42* | 55* | 57* | 50* | 39* | 42* | 57* |  |
|  | **GP** |  | **75** | **92** | **90** | **85** | **63** | **85** | **90** | **78** | Additive |
| van de Vlekkert [21] | DM | 23 | 34 | 24 | 37 | 94 | 64 | 31 | 14 | 72 |  |
|  | OM | 12 | 25 | 33 | 30 | 75 | 61 | 35 | 13 | 60 |  |
|  | NAM | 4 | 14 | 13 | 64 | 83 | 50 | 34 | 0 | 41 |  |
|  | NSM | 22 | 37 | 37 | 52 | 95 | 77 | 39 | 18 | 65 |  |
|  | **GP** |  | **70** | **90** | **80** | **80** | **78** | **70** | **80** | **80** | Additive |
| **INQOL** |  |  | **Fatigue** | **Weakness** | **Pain** | **Emotion** | **Social** | **Independance** | **Activity** | **Body image** | **Locking** |
| Rose [17] | PM/DM | 19 | 58 | 48 | 70 | 50 | 30 | 40 | 42 | 43 | 43 |
|  | IBM | 24 | 55 | 64 | 46 | 41 | 33 | 55 | 58 | 56 | 31 |

Legend: WHOQOL-BREF World Health Organization Quality of Life – BREF, SIP Sickness Impact Profile, NHP Nottingham Health Profile, SF-36 Medical Outcomes Study 36-items Short Form, INQOL Individualised Neuromuscular Quality of Life Questionnaire, PM polymyositis, DM dermatomyositis, IBM inclusion body myositis, GP general population, PF physical functioning, PR physical role, BP bodily pain, GH general health, VT vitality, SF social functioning, ER emotional role, MH mental health

‡ clinical PM without skin, elevation of creatinine kinase> 2-fold the normal limit or necrotizing myopathy

£ The scores provided by Armadans et al. were not on a scale of 0-100 as proposed by the tool’s manual and therefore cannot be compared to normative data.

* significant difference from general population (p<0.05) indicated when provided by the authors

§ significant difference between subsets (p<0.05) indicated when provided by the authors

†110 patients with BP scores
