# Supplementary material for: Health-Related Quality of Life (HRQoL) in Idiopathic Inflammatory Myopathy: A Systematic Review
Source: PLoS One. 2016 Aug 9;11(8):e0160753. doi: 10.1371/journal.pone.0160753 (PMC4978480; doi:10.1371/journal.pone.0160753)
Supplement: S4 Table — (DOCX) [file pone.0160753.s005.docx]

**S4 - Impact of clinical characteristics on HRQoL**

| **Study** | **HRQoL measure** | **Clinical data** | **Disease activity / damage measures** | **Significant findings** | | | | | |
| --- | --- | --- | --- | --- | --- | --- | --- | --- | --- |
| Armadans [12] | WHOQOL-BREF | Disease subsets  Disease duration  Age  Arthritis  Dysphagia  ILD  Heart involvement  Malignancy  Antibodies (anti-Jo1, anti-TIF1γ, anti-Mi2, anti-SRP, anti-Ro 52/60, anti-La, anti-PM-Scl, anti-RNP, anti-sm)  Treatment  Creatinine kinase (CK) | Physician Global Assessment of Disease Activity / Damage  MYOACT  MITAX  MDI  MMT8 | Older subjects had lower scores in the psychological domains (no data reported). CK level at disease onset negatively correlated with environment domain  (*r*=-0.323;p<0.05). Dysphagia associated with poorer physical (3.05 vs. 3.27;p<0.05) and environment (3.45 vs. 3.73;p<0.05) domain scores (difference did not remain after statistical adjustment). DM subjects showed higher physical scores (3.2 vs. 2.9, p=0.048) (difference disappeared after multiple comparisons). Significant Pearson correlation between disease activity and damage scores and QoL domains shown below. Small correlation 0.1; weak 0.3; large 0.5 (*p<0.05, **p<0.01, ***p<0.002). | | | | | |
|  |  |  |  |  | **Physical** | | **Environment** | | **Psychological** |
|  |  |  |  | PGA - activity | -0.290* | | -0.372** | |  |
|  |  |  |  | MYOACT | -0.282* | | -0.451*** | |  |
|  |  |  |  | MITAX | -0.321* | | -0.458*** | |  |
|  |  |  |  | MMT8 | 0.407*** | | 0.312* | |  |
|  |  |  |  | PGA - damage | -0.315* | | -0.272* | | -0.278* |
|  |  |  |  | MDI | -0.328** | | -0.268* | |  |
| Bronner [10] | SIP | Disease subsets  Antibodies (anti-Jo1, anti-synthetase, anti-Mi-2, anti-SRP)  Treatment | Rankin scale  Medical Research Council score (MRC) | Of the 37 (34%) subjects with a Rankin score of 0-1 (no disability) at follow-up, 100% had normal strength, but 23 (62%) had abnormal physical SIP score (>1.6%). All subjects with normal physical SIP score had normal strength and no (n=14) or mild (n=2) disability by the Rankin score. Myositis-specific antibodies (MSA) data shown below. | | | | | |
|  |  |  |  |  | | MSA (n) | | phSIP >1.6% n (%) | |
|  |  |  |  |  | | Anti-Jo1 (14) | | 13 (93) | |
|  |  |  |  |  | | Anti-synthetase (6) | | 6 (100) | |
|  |  |  |  |  | | Mi-2 (20) | | 16 (80) | |
|  |  |  |  |  | | SRP (3) | | 3 (100) | |
|  |  |  |  |  | | No MSA (56) | | 46 (82) | |
| Chung [11] | NHP | Disease subset | None | See Table 3. | | | | | |
| Goreshi [14] | SF-36 | Disease subsets  Pruritus / Pain (VAS) | CDASI  DLQI  Skindex-29+3  Patient Global Assessment  HAQ-DI | No data provided concerning correlation between skin severity, pruritus or pain and HRQoL measured by the SF-36. Skindex-29+3 and CDASI scores weakly correlated (r=0.2729, p<0.05) suggesting poorer QoL scores with increased cutaneous severity. Moderate to strong correlations were found between pruritus scores and Skindex-Symptom (r=0.6015, p=0.001), Skindex-Function (r=0.4552, p=0.0036) and DLQI (r=0.3477, p=0.0009). | | | | | |

**S4**

| Ponyi [15] | SF-36 | Age at diagnosis  Delay in diagnosis  Gender  Disease subsets  Disease course  Follow-up duration  Presence anti-Jo1  CK level at diagnosis  Arthralgias / arthritis  Glucocorticoid complications  Raynaud  Initial treatment  Work status | MMT  HAQ-DI | MMT scores were strongly negatively correlated with physical functioning (r=-0.57, p<0.001), moderately with role functioning (r=-0.34, p=0.001) and weakly with bodily pain (r=-0.27, p=0.01). Strong negative correlations were also found between the HAQ-DI scores and physical functioning (r=-0.71, p<0.001), role functioning (r=-0.52, p<0.001), bodily pain (r=-0.52, p<0.001). Moderate correlation reported for role emotional (r=-0.42, p<0.001) domains. | | | |
| --- | --- | --- | --- | --- | --- | --- | --- |
|  |  |  |  | **Domains** | **Significant predicting factor** | **Coefficient** | **p-value** |
|  |  |  |  | **PF** | Constant loss of muscle strength  Chronic disease course  Female  Follow-up < 60 months  Arthralgias  Osteoporosis | 69.1  -11.4  -7.9  15.9  -7.9  -10.2 | 0.000  0.015  0.026  0.000  0.020  0.002 |
|  |  |  |  | **RF** | Constant loss of muscle strength  Female  Follow-up <60 months | 76.3  -9.0  14.5 | 0.000  0.047  0.002 |
|  |  |  |  | **BP** | Constant loss of muscle strength  Female  Follow-up <60 months  Arthralgias  Compression fractures or AVN | 39.8  -15.5  -14.2  -11.6  -23.9 | 0.000  0.000  0.001  0.005  0.002 |
|  |  |  |  | **GH** | Constant loss of muscle strength  Overlap myositis  Female | 61.7  -5.1  5.6 | 0.000  0.006  0.001 |
|  |  |  |  | **VT** | Constant loss of muscle strength  Follow-up 85-120 months | 58.4  -6.7 | 0.000  0.017 |
|  |  |  |  | **SF** | Decreased muscle strength  Follow-up 61-84 months  Arthralgias | 61.1  7.1  -5.68 | 0.000  0.046  0.042 |
|  |  |  |  | **RE** | Constant loss of muscle strength  Follow-up < 60 months  Presence anti-Jo1  Osteoporosis | 69.8  13.2  -18.8  -12.4 | 0.000  0.013  0.032  0.015 |
|  |  |  |  | **MH** | Constant loss of muscle strength  Age  Initial treatment  Osteoporosis | 56.9  0.24  7.82  -5.8 | 0.000  0.026  0.024  0.028 |

**S4**

| Regardt [16] | SF-36 | Gender  Disease subsets | Grip strength  Hand mobility | Moderate to strong negative correlation between grip-force and vitality (r=0.480; p=0.05) and mental health (r=0.527;p=0.05) domains in women. |
| --- | --- | --- | --- | --- |
| Rose [17] | INQOL  SF-36 | Disease subsets  Hospital Anxiety and Depression Scale (HADS)  Illness Perception Questionnaire (IPQ-R) | HAQ | PM/DM subjects had higher level of anxiety measured by HADS (mean 10.00; sd 4.2). |
| Sadjadi [20] | SF-36 | Age  Disease duration  Beck Depression Inventory (BDI) | Amyotrophic Lateral Sclerosis-Functional Rating Scale (ALS-FRS)  MMT8  Timed stand  Time walk | Supplementary data in web tables no longer available.  MMT, timed stand, time walk and ALS-FRS scores were reported as strongly correlated with the physical domains of the SF-36. Timed walk also moderately correlated with social functioning and role emotional domains. ALS-FRS scores moderately correlated to role physical, vitality and social functioning.  BDI scores significantly correlated with every SF-36 domains except role emotional. The association found between disease severity and QoL was reduced when depression was included as a mediating variable (mediated between 1 and 14% of the effect of disease activity). |
| Sultan [18] | SF-36 | Disease course | CK | Subjects with chronic progressive course had significantly increased bodily pain than those with relapsing-remitting course (p<0.05). Correlation coefficient not provided. |
| van de Vlekkert [21] | SF-36 | Disease subsets  Disease duration  Disease course  Treatment | Modified Rankin scale | Subjects with a Rankin score of 0 had significantly better SF-36 score than those with a score of 3 in physical functioning (100 vs. 32), role physical (100 vs. 18), bodily pain (96 vs. 50), vitality (96 vs. 38) and general health (70 vs. 30) (p-value not reported).  Subjects with 2 or more relapses had lower SF-36 scores in physical (p=0.001), vitality (p=0.021) and general health (p=0.034) domains than those with a monophasic disease. |

Legend: WHOQOL-BREF World Health Organization Quality of Life – BREF, SIP Sickness Impact Profile, NHP Nottingham Health Profile, SF-36 Medical Outcomes Study 36-items Short Form, INQOL Individualised Neuromuscular Quality of Life Questionnaire, QoL quality of life, PM polymyositis, DM dermatomyositis, ILD interstitial lung disease, MYOACT Myositis Disease Activity Assessment, MITAX Myositis Intention-to-Treat Activity Index, MMT8 manual muscle testing, MDI Muscle Damage Index, PGA – activity Physician Global Assessment of Disease Activity, PGA – damage Physician Global Disease Damage Assessment, VAS Visual Analog Scale, HAQ-DI Health Assessment Questionnaire – Disability Index, CDASI Cutaneous Dermatomyositis Area and Severity Index, DLQI Dermatology Life Quality Index, PF physical functioning, PR physical role, BP bodily pain, GH general health, VT vitality, SF social functioning, ER emotional role, MH mental health
